# Supplementary material for: Nutritional status and associated factors among hospitalized adult patients in comprehensive specialized hospitals of Western Ethiopia
Source: PLoS One. 2026 May 15;21(5):e0348796. doi: 10.1371/journal.pone.0348796 (PMC13178860; doi:10.1371/journal.pone.0348796)
Supplement: S1 File — (DOCX) [file pone.0348796.s001.docx]

**ANNEXES**

**Annex I: Participant information sheet (English Version)**

**Wollega University, Institute of Health Sciences, Department of Public Health**

**Graduate Studies**

**Introduction**

Dear participant: Good morning/afternoon. My name is _------------_ and this questionnaire is prepared for a thesis research project undertaken by Birtukan Kebede (PhD student) from Wollega University Institute of health sciences on a topic entitled “Malnutrition at Admission among Adult Patients in comprehensive Specialized Hospitals of East Wollega zone, Western Ethiopia.” The aim of the study is to determine the prevalence of malnutrition and to identify factors associated among hospitalized adult patients. Therefore, in this study, you will be selected as a participant and before getting your consent, you need to know all necessary information related to the study which will be detailed as follows.

**Benefits and risk of the study**:

**Benefits:** Your participation in the study does not grant you payment or has no any special privilege to you. But your responses to the following questions are beneficial to you and other hospitalized patients as input in minimizing the magnitude of malnutrition and its effect on length of hospital stay as well as to magnify the role of hospital nutrition in improving quality of care so that recommendations will be made to responsible organizations or bodies to fill those identified gaps.

**Risks**: The study will be conducted through interviews, by measuring your height, weight. You are being asked for a little of your time, a maximum of 30 minutes, to help us in this study. There is no possible risk associated with participating in this study.

**Confidentiality**: Your name will not be written in this form and any information you tell us will not be disclosed to third party. Your participation is voluntary and you are not obligated to answer any question you do not wish to answer. If you feel discomfort with the question, it is your right to drop it any time you want. I would greatly appreciate your cooperation in advance.

Are you satisfied with the information provided so far?

1. Yes………………………….. Continue to the next page

2. No …………………………… I won’t participate

**Annex II: Consent form (English Version)**

In undersigning this document, I am giving my consent to participate in the study entitled as “Malnutrition at Admission among Adult Patients in comprehensive Specialized Hospitals of East Wollega zone, Western Ethiopia.” I have been informed the purpose of this study and I understood that participation in this study is entirely voluntarily. I have been told that my answers to the questions will not be disclosed to anyone else and no reports of this study ever identify me in any way. I have also been informed that my participation or non-participation or my refusal to answer questions will have no effect on me. I understood that participation in this study does not involve risks.

Respondent’s signature_________________________________

**001: Interviewer’s** Name_________________________Signature:___________Date:__________

002: Participant’s ID__________________

003: Supervisor: name___________ Sign_________

004: Date of Interview ________________

Time of interview: Started_____________

Completed___________

Facility name ____________________

Facility ward/admission unit ____________________

**Annex II: English version Questionnaire**

**Introduction**

**Type I: Questionnaires prepared to collect the socio-demographic factors,** **Substance uses related factors Among Adult Patients Admitted in Referral and Specialized Hospitals of East Wollega Zone through client interview.**

| **Facility name ____________________** | | | |
| --- | --- | --- | --- |
| **Facility code ____________________** | | | |
| **Client code ___________________** | | | |
| **Client medical Registration Number ________________________** | | | |
| **Part I: Socio-demographic information’s of the study participants** | | | |
| **S/No** | **Questions** | **Coding/Response Categories** | **Skip to** |
| **Q101** | What is your gender? | - Female …………………………..1 - Male ………………….……...….2 |  |
| **Q102** | How old are you now? | Age in completed years ---------------------- |  |
| **Q103** | What is the highest level of school you attended? | - unable to read and write--...................1 - Primary………………….……….......2 - Secondary………...………….............3 - high school …………………….…....4 - College/ or diploma………….……...5 - University degree …………………...6 |  |
| **Q104** | What is your Marital status? | - Unmarried/Single……….……….1 - Married ……..…………...............2 - widowed ………………………...3 - divorced………………………….4 - separated -----------------------------5 |  |
| **Q105** | What is your ethnicity? | - Oromo……………………………1 - Amara…………………………….2 - Tigre……………………………...3 - Gurage…………………………....4 - Others (specify)…………………..5 |  |
| **Q106** | What is your religion? | - Muslim ……………………..…....1 - Orthodox ………..……………….2 - Protestant ……………..………...3 - Catholic………………..………...4 - Others (specify)………..…...........5 |  |
| **Q107** | What is your occupation? | - Civil Servant ……………………1 - Farmer …………………………..2 - Merchant………………………...3 - Daily Laborer……………………4 - Housewife ………………………5 - Private employ…………………..6 - Others (specify)………………….7 |  |
| **Q108** | What is your income? | - Birr.……………………….……1 - No income --------------------------2 | Q110  If no |
| **Q109** | How much is your monthly income (ETB)? | ----------------------- Birr |  |
| **Q110** | Where is your Place of residence? | - Urban……………………………1 - Rural…………………………….2 - Semi urban……………………...3 |  |
| **Q111** | How is your living arrangement? | - Living alone…………………….1 - Living with a partner……...........2 - Living with a parent/s…………..3 - Others (specify)……...…………4 |  |
| **Part II: Substance uses related information’s of the study respondents** | | | |
| **Q201** | Have you ever smoked cigarettes? | - Yes……………………………….1 - No………………………………..2 | Q203 if no |
| **Q202** | If yes, are you currently smoking? | - Yes……………………………….1 - No……………….…..…….……..2 |  |
| **Q203** | Have you ever drunk alcohol? | - Yes………….……..…………….1 - No.………………..……………..2 |  |
| **Q204** | If yes, are you currently drinking alcohol? | - Yes……………………………….1 - No………………………………..2 |  |
| **Q205** | If yes, How often do you have a drink containing alcohol? | - Monthly or less ………………….1 - 2 to 4 times a month……………..2 - 2 to 3 times a week……………...3 - 4 or more times a week………….4 |  |
| **Q206** | Have you ever chewed the khat? | - Yes……………………………….1 - No………………………………..2 | If, no Part II |
| **Q207** | If yes, how frequent do you chew khat? | - Frequently………………………..1 - Twice a week…………………….2 - Occasionally…………………….3 |  |
| **Part III: Questionnaires used to assess the base-line clinical information; the impact of malnutrition on clinical outcomes of adult patients admitted in specialized hospitals of East Wollega Zone. This was done through client interviews and reviewing the clients documents.** | | | |
| **Q301** | What is patients Admission ward? | - Medical …………………...........1 - Surgical………………………….2 - Orthopedic……………………….3 |  |
| **Q302** | What is patient’s admission primary diagnosis? | - Congestive heart failure………….1 - Gastrointestinal……...……...........2 - Genitourinary…………………….3 - Upper Respiratory….……………4 - Lower Respiratory……………….5 - Neurology………………………..6 - Musculoskeletal………….………7 - Anemia…………………………..8 - Hematology ……….. .……..........9 - Trauma …………………………10 - Others (specify).……………….11 |  |
| **Q303** | Please specify admission primary diagnosis (review the chart and specify) | ---------------------------------------- |  |
| **Q304** | Admission secondary diagnosis  (comorbidities) | - Yes ……………………………...1 - No …………………………..…..2 | If no, skip to Q 306 |
| **Q305** | If yes to Q304, specify admission Secondary diagnosis | - ………………… |  |
| **Q306** | Is there any clinical evidence /dx of infection at the time of  admission?(please review the chart) | - Yes ………………………………1 - No ……………………………….2 | 308 if no |
| **Q307** | Please specify the type of infection (please review the chart) | -------------------- |  |
| **Q308** | Are you currently taking any Drugs? | - Yes ……………………………...1 - No ……………………………....2 | If no, skip to Q 310 |
| **Q309** | How many different drugs are you  Taking per day? | - ------------------------ |  |
| **Q310** | Is there any history of previous  Admission in the past five years? | - Yes …………………………...….1 - No …………….............................2 | If no, skip to Q 311 |
| **Q311** | If yes, please specify the number of  admissions | - -------------------------------- |  |
| **Q312** | Is there any history of previous  Surgery in the past five years? | - Yes ……………………...........….1 - No …………….............................2 |  |
| **Q313** | Did you use a diverse diet at home? | - Yes……………………………….1 - No ……………………………….2 |  |
| **Q314** | Did you use a diverse diet during admission? If Yes which one? | - Yes……………………………….1 - No ……………………………….2 | If Yes, |
| **a** | starchy staples (grains, white roots, tubers, and plantains), | - Yes……………………………….1 - No ……………………………….2 |  |
| **b** | legumes (beans, peas, and lentils), | - Yes……………………………….1 - No ……………………………….2 |  |
| **c** | nuts and seeds, | - Yes……………………………….1 - No ……………………………….2 |  |
| **d** | dairy products, | - Yes……………………………….1 - No ……………………………….2 |  |
| **e** | flesh foods (meat, poultry, and fish), | - Yes……………………………….1 - No ……………………………….2 |  |
| **f** | eggs, | - Yes……………………………….1 - No ……………………………….2 |  |
| **g** | dark green leafy vegetables, | - Yes……………………………….1 - No ……………………………….2 |  |
| **h** | vitamin A-rich fruits and vegetables, | - Yes……………………………….1 - No ……………………………….2 |  |
| **i** | other vegetables, and | - Yes……………………………….1 - No ……………………………….2 |  |
| **j** | other fruits | - Yes……………………………….1 - No ……………………………….2 |  |
| **Q315** | Did you receive nutritional support during your hospital stay? | - Yes ………………………………1 - No …………….............................2 |  |

**Part IV. Questionnaires used to assess nutritional status at admission using Subjective global assessment (SGA) related information’s of the study respondents**

**Instruction፡ Now I am going to ask you questions about your (patient’s) nutritional status**

| **No.** | | **Questions** | **Coding category** | **Remark (skip)** |
| --- | --- | --- | --- | --- |
| **Q401** | | Have you (patient) lost weight in the past 6 Months? | - Yes …………………….1 - No ……………………..2 |  |
| **Q402** | | How much weight has changed in the past 6 months? | - Weight loss (kg)----------- |  |
| **Q403** | | Approximately how much weight has changed in the past 6 months? | - <5% loss or weight stability…………………1 - 5-10% loss without stabilization or increase...2 - >10% loss and ongoing...3 |  |
| **Q404** | | If above not known, has there been a subjective loss of weight during the past six months? | - None or mild - Moderate - Severe |  |
| **Q405** | | Weight change past 2 weeks* Amount (if known) | - Increased - No change - Decreased |  |
| **Q406** | | What was your (patient’s) usual weight before the disease? | ……..……………. Kg |  |
| **Q407** | | What is current patient’s weight? (Please measure the current weight of the respondent) | ………..……………. Kg |  |
|  | |  |  |  |
| **Q408** | | Is there any change in your (patient)dietary  intake after the disease? | Yes…………………………… 1  No……………………………..2 | **To Q 409 & 410** |
| **Q409** | | What kind of dietary intake change  is there? | Borderline or improving or declining…………………..…...1  Poor and decreasing……….…..2  Starvation, unable to…………..3 |  |
| **Q410** | | What type of diet are you (patient) currently taking? | Suboptimal liquid diet………....1  Full liquid diet……….…….......2  Starvation……...........................3 |  |
| **Q411**  **Q412** | | Which gastrointestinal symptoms has persisted for the past 2 weeks and more?  (Experiencing symptoms affecting oral intake)  **Multiple Response is possible**  **How server gastrointestinal symptoms?** | 1.   - Nausea……………...….2 - Vomiting…………....…3 - Diarrhea…………….....4 - Anorexia….…………...5 - Pain on eating - Feels full quickly - Dental problems - Dysphagia - Constipation   2.   - None - Intermittent/mild/few - Constant/severe/multiple   Symptoms in the past 2 weeks*   - Resolution of symptoms - Improving - No change or worsened |  |
|  | |  |  |  |
| **Q413** | | Is there a change in your (patient) functional capacity? | - Yes………………….…1 - No……….…………….2 | **To Q 413** |
| **Q414** | | How is the type of your (patient) functional capacity change? | - Working sub optimally…1 - Ambulatory……….……2 - Difficulty with ambulation/normal activities…….................3 - Bed/chair-ridden……....4 |  |
| **Q415** | | How long your (patient) functional capacity changed? (Reduced capacity) | - duration of change: _____ |  |
| **Q416** | | How is your (Patient) metabolic demand (stress)?  **filled by the data collector** | - No stress ……………….1 - Low stress…...…………2 - moderate stress…………3 - High stress..………….…4 |  |
| **Physical assessment** | | | | |
| **Q417** | **Loss of Subcutaneous (SC) fat (triceps,)/** **Loss of body fat**  **Remark received**   - None (Fingers don’t touch) - Low to moderate (Fingers nearly meet) - Severe (Fingers touch) | | - None …………………...1 - Mild/Moderate…………2 - Severe…………………..3 |  |
| **Q418** | **Is there muscle wasting in the clavicle area?**  **Remark**   - **(Low to moderate)**   Muscle loss not presenting; visible, but prominent in females  **In males**: a portion of the clavicle is  visible  **In females**: the clavicle is prominent deltoid and chest muscle is still intact   - **(Severe)**   Evident protrusion | | - None …………………..1 - Low to moderate….........2 - Severe………………….3 |  |
| **Q419** | **Is there Edema/Ascites?**  (Please review the chart of the  patient)  **(Remark)**   - Mild edema…. Localized to lower extremities (Ankle, pedal, tibial) - Severe edema …Generalized edema | | - No-edema ………………1 - Mild edema…………….2 - Severe edema…………..3 |  |

**Part V. Questionnaires used to assess the length of stay, hospitalization- related outcomes, and anthropometric data of the study respondents**

**Anthropometric data**

| **No** | **Question** | **Coding category** | **Remark**  **(Skip)** |
| --- | --- | --- | --- |
| **Q501** | Date of admission (dd/mm/yyyy) E.c | **_____/ _____/__________** |  |
|  | |  |  |
| **Q502** | What is the weight in kilograms at admission? | **_______ kg** |  |
| **Q503** | What is the height in meters at admission? | **_______ m** |  |
| **Q504** | Date of Discharge (dd/mm/yyyy) E.C | ________/ ______/ _______ |  |
| **Q505** | What is the weight in kilograms at discharge? | **_______ kg** |  |
| **Q506** | What is the height in meters at discharge? | **________m** |  |
| **Hospitalization related Outcome** | | | |
| **Q507** | Is there any clinical evidence or diagnosis of infection **during hospitalization (Nosocomial infection)**?  (**please review the chart**) | - Yes ……………………….1 - No ………….......................2 |  |
| **Q508** | Was he/she readmitted within the past 30 days after discharged | - Yes ……………………….1 - No ………….......................2 |  |
| **Q509 b** | Is there any evidence Poor wound healing? | - Yes ……………………….1 - No ………….......................2 |  |
| **Q510** | Is he/she unplanned transfer to CCA (Critical Care Areas)? | - Yes ……………………….1 - No ………….......................2 |  |
| **Q511** | Is he/she died (lost life) in-hospital? | - Yes - No |  |
