# Supplementary material for: Nutritional status and associated factors among hospitalized adult patients in comprehensive specialized hospitals of Western Ethiopia
Source: PLoS One. 2026 May 15;21(5):e0348796. doi: 10.1371/journal.pone.0348796 (PMC13178860; doi:10.1371/journal.pone.0348796)
Supplement: Attachment — Submitted filename: As reviewer.docx [file pone.0348796.s003.docx]

Manuscript Title:

*Nutritional status of hospitalized adult patients in comprehensive specialized hospitals of Western, Ethiopia*

Reviewer Name

Samuel Abate Walda (BSc, MSc in adult health nursing, lecturer)

**Overall Recommendation:-Major Revision**

**1. Summary of the Study**

The authors conducted a facility-based cross-sectional study assessing the prevalence of malnutrition and associated factors among 927 adult hospitalized patients in two comprehensive specialized hospitals in Western Ethiopia. Nutritional status was evaluated using the Subjective Global Assessment (SGA). The study reports a high burden of malnutrition (58.9%) and identifies several predictors, including age, sex, education, comorbidities, and dietary diversity.

**2. General Assessment**

The topic is important, timely, and relevant to global hospital nutrition and public health in low-resource settings. The manuscript is generally well organized, the methods are mostly sound, and the findings are potentially valuable for clinicians and policymakers.

However, major methodological clarifications and corrections are needed before the paper can meet PLOS ONE standards for scientific rigor. Several issues exist regarding sample size justification, statistical coding errors, and variable definitions, clarity of SGA assessment procedures, inconsistent reporting, and figure/table formatting.

With substantial revision, the manuscript has the potential for publication.

**3. Major Concerns**

**1. Sample size mismatch (critical)**

The authors report an intended sample size of 1,106, yet only 927 respondents were included (84% response rate).

However, they do not discuss:

Why the response rate was low.

How non-response may affect representativeness.

Whether power was recalculated or compromised.

**Action required:**
Provide a justification for the final achieved sample and discuss implications of non-response.

**2. Coding error in the statistical analysis section**

The manuscript states:

“coded ‘0’ for well-nourished… ‘1’ for moderately malnourished… and ‘2’ indicating middle category, and ‘2’ for severely malnourished.”

There is a clear duplicated value (‘2’) for two different categories, which is a serious methodological problem.

**Action required:**
Correct the coding scheme and confirm that model outputs are based on correctly ordered categories.

**3. Use of SGA requires more detail to ensure reproducibility**

The SGA assessment process is insufficiently described. Examples of missing details include:

Who performed SGA? Were they trained nutritionists or only nurses?

Was inter-rater reliability assessed?

Was SGA validated in similar Ethiopian hospital settings?

How were subjective components standardized?

**Action required:**
Provide clear description of SGA training, standardization, quality control, and inter-observer agreement.

**4. Dietary diversity measurement inconsistencies**

Two different descriptions of dietary diversity appear:

One uses 11–12 food groups (including coffee/tea, sugar, oils, etc.).

Another uses the FAO MDD-W 10-food group standard.

This inconsistency undermines the validity of the dietary diversity variable.

Action required:
Clarify which dietary diversity tool was used, ensure alignment with valid guidelines, and re-analyze if needed.

**5. Interpretation of AOR for residence appears reversed**

The result states:

“Urban residence AOR = 0.49… lower odds of experiencing moderate or severe malnutrition compared to semi-urban”

But earlier, the authors claim:

“Urban residence was associated with undernutrition”

This is contradictory.

Action required:
Clarify reference category, interpretation, and correct contradictory statements.

**6. Confounder selection justification is missing**

PLOS ONE requires transparency in model building. The authors do not describe:

How variables were selected for the multivariable model (p < 0.25? theoretical framework?)

Whether multicollinearity was assessed.

Whether any variables were excluded due to collinearity or small cell sizes.

**Action required:**
Explain confounder selection strategy and report tests for multicollinearity.

**7. Ethical issues regarding consent**

The statement “thumbprint written consent” is ambiguous. Clarification needed:-

Was the thumbprint used on a written consent form?

Who served as witness?

Were illiterate participants informed through a standardized script?

**8. Literature review has inaccuracies or outdated references**

The section is overly long and contains inaccuracies:

Some percentages do not match cited papers.

Narrative occasionally repeats findings unnecessarily.

Referencing style is inconsistent with PLOS ONE format.

**Action required:**
Revise the introduction for conciseness, accuracy, and updated references.

**4. Minor Comments**

Abstract

Grammar and flow need slight refinement.

Methods should explicitly mention analysis software once (avoid repetition).

**Tables**

Table 1: "Secondary" and "Secondary/High school" categories appear redundant.

Table 2: Some parentheses are mismatched, and percentages do not always sum logically.

Table 3: Occupational categories unclear; “others” is too vague.

Figures

Figure 1 is referenced but not provided in the document

**Writing and clarity**

Several grammatical inconsistencies require language editing.

Discussion occasionally restates results instead of interpreting them.

Referencing

Should follow PLOS ONE numeric style consistently.

DOIs should be included where available.

**5. Strengths of the Manuscript**

Large sample size compared to most studies in Ethiopia.

Important clinical topic with public health significance.

Use of SGA (a validated tool).

Appropriate use of ordinal logistic regression (assuming corrections are made).

Clear presentation of ethical approvals and data availability.

**6. Specific Questions for Authors**

How “inadequate dietary diversity” was operationally defined?

Were any patients excluded due to inability to complete SGA reliably?

Were acute infection markers (e.g., fever) considered confounders in the model?

Did the study consider hospital food services or meal frequency?

Could selection bias exist since emergency patients were excluded?

**7. Recommendation to the Editor**

Major Revision

The manuscript addresses a relevant public health issue and contains potentially important findings, but substantial methodological clarifications and corrections are needed to ensure scientific rigor and reproducibility. Once these major issues are addressed, the manuscript may be suitable for reconsideration.
