# Supplementary material for: Nutritional status and associated factors among hospitalized adult patients in comprehensive specialized hospitals of Western Ethiopia
Source: PLoS One. 2026 May 15;21(5):e0348796. doi: 10.1371/journal.pone.0348796 (PMC13178860; doi:10.1371/journal.pone.0348796)
Supplement: Attachment — Submitted filename: Reviewers comment.docx [file pone.0348796.s004.docx]

Dear Author/s,

Thank you for conducting research on the nutritional status of hospitalized adult patients in comprehensive specialized hospitals of Western Ethiopia. It gives a good overview of the nutritional status of hospitalized adult patients in western Ethiopia. In your research paper I noticed the following points, which I am not clear on.

1. In the Abstract section, on line 40. You wrote “predictors terminology,” but in the results section you wrote association factors. Please use consistent terminology.
2. Also, your sample size is inconsistent. 1106 patients participated in the study in the abstract section, but 927 patients participated in the result section. How could it be a different figure?
3. On line 64, what is hospital malnutrition? That means patients developed undernutrition due to their hospitalization? It is better if replaced by malnutrition among adult patients admitted to the hospitals.
4. I am not satisfied with the conclusion section! Only you rewrote your results in the conclusion section. It is better if you add the consequence or impact of undernutrition.
5. On line 90, the first sentence of the paragraph needs a citation.
6. Line 110, regarding the gap of the study, I am not satisfied. Studies that were conducted in Ethiopia have limitations? In terms of variable or study design? Or did you because they are few in number? What were their gaps?
7. line 125, you stated, “According to the Oromia Regional Health Office, Zonal Health Office, and Health Information Management System of each hospital, these hospitals provide health care services for more than 3.1 million people in the East Wollega zone, 1.5 million people in West Shewa, and 1.2 million people in the Buno Bedele zone.” Do you mean that each hospital has its own separate catchment population? Is it possible? Please clarify!
8. Line 130, gynecology ward: was it included or excluded?
9. line 132 you stated, “…..However, patients with physical disabilities such as kyphosis, scoliosis, and limb defects including deformities, as well as pregnant patients, unconscious or comatose patients, obstetrics patients, surgical emergency patients, and patients to be discharged within 72 hours were excluded from the study.” Is there a difference between pregnant and obstetric patients? Why did you mention obstetrics patients separately?
10. If you excluded patients who discharged within 3 (72 hrs.) days, how could you get this sample size? What was the hospital’s average length of hospital stay?
11. In lines 154, 155, and 156, the assessment was made by whom? Nurse or physician, but you didn't include physician in the data collection procedure. So justify your data quality?
12. Have you excluded patients who have no lower extremities (amputated patients) in the orthopedics ward?
13. Line 193 you have did pretest. But you didn’t state the hospital. At the same hospital?
14. Line 301 Please add CI
15. line 318 in the discussion section, you stated, “Nevertheless, the results of the current study is found to be lower than results of a few studies reported from Iran (23.92%) (20), Korea (22.0%) (21), and the university hospital in Bobo-Dioulasso, Burkina Faso (32.4%) (22).” It needs justification
16. Line 365, in the conclusion section. Do you think only hospital-based intervention could solve the malnutrition problem? I think this undernutrition prevalence was not due to hospitalization. Why didn’t you recommend community-based intervention?
17. It is better if you add recommendations like strengthening nutritional policy at Ethiopian hospitals.
