# Supplementary material for: Nutritional status and associated factors among hospitalized adult patients in comprehensive specialized hospitals of Western Ethiopia
Source: PLoS One. 2026 May 15;21(5):e0348796. doi: 10.1371/journal.pone.0348796 (PMC13178860; doi:10.1371/journal.pone.0348796)
Supplement: Attachment — Submitted filename: Comments by GF.docx [file pone.0348796.s005.docx]

Dear reviewer, thank you for inviting me for reviewing the paper. I congratulate the authors for excellent job. It is written paper, and the are is also very wonderful. I have forwarded few comments that researchers can include for the better of the work.

Study title: **Nutritional status of hospitalized adult patients in comprehensive specialized hospitals of Western, Ethiopia.**

Comment: The author comes with excellent area which is timely and seeks immediate action. However, the title and the study objective don’t inline. I wonder if the author re-writes the title as “Nutritional status and associated factors of hospitalized adult patients in comprehensive specialized hospitals of Western, Ethiopia”

1.1. **Background**

**Comment:** The study is cross-sectional study and the last sentence of abstract should have to avoid ‘predictor’ because it better fit for cohort study and it is difficult address predictor using cross-sectional study design.

1.2. **Method**

How was the proportional Samples being proportionally allocation to each hospital based on their patient population size was made? Was it ward based? Some period admission? or what else. It has to identified.

1.3. Conclusion

I hope the first sentence of this sub-section is to mean. “Hospitalized patient malnutrition….”

It will be good if the sub-section also involves the recommendation.

Discussion of is well written. However, I wonder if the researcher add implication for each result like: for practice, policies, and future research.

What are the strength and limitation of current study so that readers can use it effectively.

What are the key recommendation of the researchers from current finding?

I wonder if researchers add declaration section for the manuscript as per the journal guideline.
