# Supplementary material for: Nutritional status and associated factors among hospitalized adult patients in comprehensive specialized hospitals of Western Ethiopia
Source: PLoS One. 2026 May 15;21(5):e0348796. doi: 10.1371/journal.pone.0348796 (PMC13178860; doi:10.1371/journal.pone.0348796)
Supplement: Attachment — Submitted filename: Comments on the manuscript_PONE-D-25-57131.docx [file pone.0348796.s006.docx]

General comments

This manuscript addresses an important and under-researched public health problem and aligns well with PLOS ONE’s scope. The study benefits from a relatively large sample size, use of a validated assessment tool (SGA), and appropriate statistical modelling (ordinal logistic regression). However, the manuscript requires substantial revision to improve clarity, internal consistency, methodological transparency, and language quality. Several inconsistencies in sample size, variable definitions. The primary focus of the study should also be clarified. The followings are important areas to be improved by the authors

**Specific Comments**

**Title**

I suggest modifying the title to: **“Nutritional status and its associated factors among adult patients admitted to …”** as the authors mentioned the results of associated factors in the results, discussions and conclusion parts.

**Abstract**

**Background**

The background and objective should be consistent. Currently, the background seems to focus on malnutrition among the general adult population rather than adult patients admitted to hospitals. It would be better to align the background with the actual study population (i.e., adult admitted patients).

**Methods**

The authors should clearly describe the characteristics of the admitted patients. Patients with different health conditions may have different nutritional statuses; therefore, specifying these characteristics is important.

**Results**

The authors report a prevalence of 24.5%, but it is unclear how this figure was classified as high or low. The result should be interpreted with reference to appropriate national or international standards or guidelines.

**Conclusion**

Some of the recommendations are not supported by the study findings. For example, the authors recommend improving hospital nutritional care plans and guidelines; however, no results indicate the absence or inadequacy of such plans. Similarly, the recommendation that “hospital-based nutrition interventions should take into account multidisciplinary approaches” raises questions, as it is unclear whether comprehensive specialized hospitals in western Ethiopia are currently providing such services, and no findings appear to address this issue.

**Introduction**

Although the authors focus on malnutrition, the introduction primarily discusses under nutrition and underweight. It would be important to discuss the broader categories of malnutrition, including under nutrition, overweight, obesity, protein-energy malnutrition **(**PEM), and other forms, and then clearly indicate which type(s) are most prevalent among the study population.

**Methods**

- The authors did not report the size of the target population.
- It is important to specify the types of adult admitted patients included in the study (e.g., medical vs. surgical wards, acute vs. chronic conditions), as nutritional status may differ across these groups.
- The proportion value used for sample size calculation should be clearly stated.
- There is inconsistency in the sampling description. The authors state that simple random sampling was used, yet enrolment continued until the desired sample size was reached. This raises concerns about whether the method was truly probability-based or closer to consecutive or quota sampling.
- The sampling frame used for simple random sampling should be clearly described.

**Ethical Considerations**

- On page 10, line 225, and the authors’ state: *“All necessary interventions were made to treat undernourished patients.”*
  This statement is unclear. Does it imply that the study included an intervention component? If not, clarification is needed.

**Results**

- Please ensure consistency in terminology. Terms such as **“**severely malnourished,” “severe malnutrition,” “sever malnourished,” “moderate malnutrition,” and “moderately malnourished” are used in this document interchangeably, which is confusing.
- The focus of the study should be clarified: is it malnutrition or under nutrition? The background and results sections appear inconsistent in this regard. Operational definitions for the main outcome variables and key terms should be provided.
- Table 2 is titled *“Proportion of under nutrition,”* yet the table content refers to *malnutrition*. These terms should not be used interchangeably without clarification.
- Since malnutrition includes both under nutrition and over nutrition, the authors should be specific and focused on the intended outcome.
- On page 15, lines 275–288, the focus shifts entirely to under nutrition, despite earlier references to malnutrition. This inconsistency requires careful revision.
- Table 3 should be clearer. The outcome variable should be explicitly shown, and results should be presented using appropriate cross-tabulations, including variables, COR, AOR, and p-values.

**Discussion**

Similar to the concerns I rose in the background, methods, and results sections, the authors also used malnutrition and under nutrition under discussion interchangeably. The discussion should clearly and consistently focus on the defined outcome variable.

**Conclusion**

The conclusion appears to focus primarily on under nutrition rather than malnutrition. The authors should clearly state the actual focus of the study and avoid using these terms interchangeably.
