# Supplementary material for: Nutritional status and associated factors among hospitalized adult patients in comprehensive specialized hospitals of Western Ethiopia
Source: PLoS One. 2026 May 15;21(5):e0348796. doi: 10.1371/journal.pone.0348796 (PMC13178860; doi:10.1371/journal.pone.0348796)
Supplement: Attachment — Submitted filename: Reviewerscomment 2N.docx [file pone.0348796.s008.docx]

Dear Author/s,

Thank you for conducting research on the nutritional status of hospitalized adult patients in comprehensive specialized hospitals of Western Ethiopia. It gives a good overview of the nutritional status of hospitalized adult patients in western Ethiopia.

To sum up, I think you may have revised and answered my comments and suggestions, but I feel there is still some work to do to mature the manuscript. Especially, there are still some grammatical and technical errors in the presentation. A further proof reading is still needed to standardize the language of presentation.
