# Supplementary material for: Nutritional status and associated factors among hospitalized adult patients in comprehensive specialized hospitals of Western Ethiopia
Source: PLoS One. 2026 May 15;21(5):e0348796. doi: 10.1371/journal.pone.0348796 (PMC13178860; doi:10.1371/journal.pone.0348796)
Supplement: Attachment — Submitted filename: Response to Reviewers.docx [file pone.0348796.s009.docx]

**Additional Editor Comments:**

1) Please thoroughly revise the entire document for typographical and grammatical errors.

- **Response:** Dear reviewer, thank you for your precious comment. We have tried all the best to review the document/manuscript for all possible typographical and grammatical errors.

2) On line 47 - Unless you have two or more distinct sample populations, use the term "sample" rather than "samples."

- **Response:** Dear reviewer, thank you for your precious comment. We have amended it in the revised version as indicated on line 39.

3) Use the past tense throughout the report and for your objectives (Line 112), except within the conclusion and recommendations sections.

**Response:** Dear reviewer, thank you for your invaluable comment. It has been amended in the revised version on line 106.

4) Provide a reference for the information used in the "Study Area" section

**Response:** Dear reviewer, thank you for your invaluable comment. It has been amended in the revised version on line 115.

5) For the methodological clarification, while data collection lasted five months, the caseload for proportional allocation was based on only three months. Please clarify how you determined the specific number of samples for each month, week, or day.

- **Response:** Dear reviewer, thank you for your invaluable comment**.** Apology for misstatement of the data collection period**.** The actual data collection period ranged from December 16, 2023 to April 16, 2024 (line38-39, 108). In this study, both the data collection period and the caseload used for proportional allocation covered the same months. The total caseload during this period was obtained from hospital records (one quarter) and used as the basis for determining the sample size distribution. First, the overall sample size was proportionally allocated to each study site according to their contribution to the total four months caseload. Then, for implementation purposes, the allocated sample for each site was further distributed across months but not disaggregated on daily and weekly basis.

6) Under the operational definition, please specify the exact diseases being studied, as not all conditions are directly relevant to your outcome variable (nutritional status).

- **Response:** Dear reviewer, thank you for your invaluable comment. The most common comorbidities observed among the patients during admission included congestive heart failure, diabetes mellitus, tuberculosis, hypertension, HIV/AIDS, and urinary tract infection which can have an association with nutritional status of patients as indicated on line 192-195 in the revised version. Moreover nutritional status is reconsidered under operational definition section.

7) The non-response rate is significantly high; please provide a clear explanation or justification for this figure

- **Response:** Dear reviewer, thank you for your invaluable comment. The probable explanation for having high non-response rate, 16% might be attributed to early discharge or patient transfer, refusals to participate due to fatigue or lack of interest, which could have contributed to such non-response rate, and an underestimation of proportion of malnutrition, resulting in slightly less response rate. This concern has been discussed as a limitation of the study in that the possibility of introduction non-response bias is more likely.

8) As "semi-urban" is not an official category in the current Ethiopian census or administrative system, please clarify its definition and criteria within your study

- **Response:** Dear reviewer, thank you for your concern on the concept of “Semi-urban” in our study. As you mentioned, in Ethiopia, it is not a strictly standardized administrative category (like urban or rural defined by the government), but it is **commonly used in research and planning** to describe areas that are **in transition between rural and urban settings.**

9) **Reviewer #2:** Dear Author/s,
   Thank you for conducting research on the nutritional status of hospitalized adult patients in comprehensive specialized hospitals of Western Ethiopia. It gives a good overview of the nutritional status of hospitalized adult patients in western Ethiopia.
   To sum up, I think you may have revised and answered my comments and suggestions, but I feel there is still some work to do to mature the manuscript. Especially, there are still some grammatical and technical errors in the presentation. A further proof reading is still needed to standardize the language of presentation.

- **Response:** Dear reviewer, thank you for your invaluable comment. We have critically evaluated the revised version for typos and grammar as much as possible.
